# Heightened COVID-19 Mortality in People With Severe Mental Illness Persists After Vaccination: A Cohort Study of Greater Manchester Residents

**DOI:** 10.1093/schbul/sbac118

**Published:** 2022-08-27

**Authors:** Lamiece Hassan, Chelsea Sawyer, Niels Peek, Karina Lovell, Andre F Carvalho, Marco Solmi, George Tilston, Matthew Sperrin, Joseph Firth

**Affiliations:** Division of Psychology and Mental Health, The University of Manchester, Manchester Academic Health Science Centre, Manchester, M13 9PL, UK; Division of Psychology and Mental Health, The University of Manchester, Manchester Academic Health Science Centre, Manchester, M13 9PL, UK; Centre for Health Informatics, Division of Informatics, Imaging and Data Sciences, The University of Manchester, M13 9PL, UK; NIHR Greater Manchester Patient Safety Translational Research Centre, The University of Manchester, Manchester, UK; Manchester Academic Health Science Centre, National Institute for Health Research Manchester Biomedical Research Centre, The University of Manchester, Manchester, M13 9PL, UK; Division of Nursing, Midwifery and Social Work, University of Manchester, Manchester Academic Health Science Centre, Manchester, M13 9PL, UK; IMPACT (Innovation in Mental and Physical Health and Clinical Treatment) Strategic Research Centre, School of Medicine, Barwon Health, Deakin University, Geelong, Victoria, Australia; Psychiatry Department, University of Ottawa, Ottawa, ON, Canada; The Ottawa Hospital, University of Ottawa, Ottawa, ON, Canada; Clinical Epidemiology Program, Ottawa Hospital Research Institute (OHRI), University of Ottawa, Ottawa, ON, Canada; Centre for Health Informatics, Division of Informatics, Imaging and Data Sciences, The University of Manchester, M13 9PL, UK; Manchester Academic Health Science Centre, National Institute for Health Research Manchester Biomedical Research Centre, The University of Manchester, Manchester, M13 9PL, UK; Centre for Health Informatics, Division of Informatics, Imaging and Data Sciences, The University of Manchester, M13 9PL, UK; Division of Psychology and Mental Health, The University of Manchester, Manchester Academic Health Science Centre, Manchester, M13 9PL, UK; Greater Manchester Mental Health NHS Foundation Trust, Manchester Academic Health Science Centre, Manchester, M13 9PL, UK

## Abstract

**Background and Hypothesis:**

Previous studies show that people with severe mental illness (SMI) are at higher risk of COVID-19 mortality, however limited evidence exists regarding risk postvaccination. We investigated COVID-19 mortality among people with schizophrenia and other SMIs before, during and after the UK vaccine roll-out.

**Study Design:**

Using the Greater Manchester (GM) Care Record to access routinely collected health data linked with death records, we plotted COVID-19 mortality rates over time in GM residents with schizophrenia/psychosis, bipolar disorder (BD), and/or recurrent major depressive disorder (MDD) from February 2020 to September 2021. Multivariable logistic regression was used to compare mortality risk (risk ratios; RRs) between people with SMI (*N* = 193 435) and age–sex matched controls (*N* = 773 734), adjusted for sociodemographic factors, preexisting comorbidities, and vaccination status.

**Study Results:**

Mortality risks were significantly higher among people with SMI compared with matched controls, particularly among people with schizophrenia/psychosis (RR 3.18, CI 2.94–3.44) and/or BD (RR 2.69, CI 2.16–3.34). In adjusted models, the relative risk of COVID-19 mortality decreased, though remained significantly higher than matched controls for people with schizophrenia (RR 1.61, CI 1.45–1.79) and BD (RR 1.92, CI 1.47–2.50), but not recurrent MDD (RR 1.08, CI 0.99–1.17). People with SMI continued to show higher mortality rate ratios relative to controls throughout 2021, during vaccination roll-out.

**Conclusions:**

People with SMI, notably schizophrenia and BD, were at greater risk of COVID-19 mortality compared to matched controls. Despite population vaccination efforts that have prioritized people with SMI, disparities still remain in COVID-19 mortality for people with SMI.

## Introduction

People with severe mental illness (SMI) die approximately 15 years younger than the general population, primarily due to the heightened morbidity and mortality from physical diseases among this vulnerable group.^1–4^ This elevated risk applies to both noncommunicable and infectious diseases and recent evidence from around the world—summarized in several recent systematic reviews and meta analyses^5–11^—has now shown that people with SMI are also disproportionately affected by COVID-19.

While people with mental illnesses appear to be more vulnerable to COVID-19,^5–11^ the extent to which disparities in outcomes apply across different diagnostic groups of mental illness, and the reasons underpinning this, is a complex and dynamic subject, not fully elucidated by research. Available evidence points towards a combination of sociodemographic factors, preexisting comorbidities, and disease related factors (eg, psychotropic medication) as possible explanatory factors contributing towards increased risks of COVID-19 related infection, hospitalization, and mortality among people with SMI.^5–8^ Despite well-conducted and thorough studies, however, gaps and methodological limitations apply to several areas. For example, a recent systematic review of 23 studies on COVID-19 in mental illness reported that only a minority adjusted for preexisting conditions.^7^ Moreover, systematic reviews and meta-analyses have adopted varying search strategies and definitions of key variables, including COVID-19 infection itself, complicating efforts to synthesize knowledge in this area.^8^

Two of the largest UK studies to date^12,13^ used the UK Biobank (UKB) cohort, a longitudinal cohort study linking primary care, hospital data, and death records for half a million older adults. These both reported higher rates of COVID-19 related hospitalization and mortality among people with mental illness, including schizophrenia, in the period prior to the UK vaccine roll-out. While considered a valuable resource, UKB participants are well known to be older, healthier and less ethnically diverse than the general population.^14^ Thus, while studies have generated some useful evidence, there remains a need to conduct research using more representative samples of the population, particularly those that include ethnically diverse groups and younger populations.

Concerns have led to calls for people with schizophrenia and certain other severe mental illnesses to be prioritized for access to vaccinations^15^; advice which has been followed by several countries, including the United Kingdom.^16^ However, given the combination of biopsychosocial factors that may affect vaccine uptake and immune response among people with mental illness,^17,18^ evidence is still needed to demonstrate whether these disparities in COVID-19 mortality risks still persist following vaccination. In England, the NHS started administering COVID-19 vaccinations on December 8, 2020, with people with SMI prioritized for vaccination alongside people aged 70 years and over on advice from an independent expert advisory committee.^19^ By April 12, 2021, all people aged over 50 years (including SMI groups) had been offered a first vaccination; second vaccinations were offered 3–12 weeks after the individual’s first dose. An early analysis of vaccination uptake nationally in England, albeit limited to patients over 80 up until March 17, 2021, showed that vaccination was initially lower among people with severe mental illnesses and learning disabilities.^20^ A more recent study by Bitan^21^ that followed up people with schizophrenia in Israel throughout the first year of the pandemic found that mortality rates declined in these populations following mass vaccination efforts, though they remained relatively higher compared to controls. The same study also noted that people with schizophrenia were less likely to be vaccinated.

In this study we aimed to examine COVID-19 related mortality among people with schizophrenia and other SMIs, compared to similar people without SMI, over a 20-month period (February 2020 to September 2021). This covered the period prior to, during, and after the initial vaccine roll-out in the United Kingdom. We also aimed to account for the influence of sociodemographic characteristics, preexisting clinical conditions, and vaccination status on the risks of COVID-19 related mortality among people with SMI. In doing so, we build upon previous studies to support enhanced understanding of the size and nature of the risks posed by COVID-19 to people with mental illness to inform public health strategies in a postvaccine world.

## Methods

### Design and Participants

We used de-identified patient data from the Greater Manchester Care Record (GMCR), a city wide, integrated digital care record containing information related to 3.2 million residents. Used by over 500 health and social care organizations to provide direct care to patients, the GMCR also includes details of primary care, hospital stay episodes, and deaths.

Using the GMCR, we compared COVID-19 mortality in three overlapping samples of patients with SMI (each with matched controls), namely people with schizophrenia, BD, and/or MDD (supplementary material - figure S1). All patients who were alive, aged 18 years and over and registered with a general practitioner in GM as of January 31, 2020 (the date of the first UK COVID-19 related death) were eligible for inclusion in the samples. Participants were followed up for up to 20 months in total; from the start of the pandemic (February 1, 2020) until September 30, 2021 or death, whichever occurred first.

This study was approved via the GMCR’s secondary uses and research governance process, which involved review against legal, ethical, and information governance criteria. A patient and public involvement (PPI) group of 14 regular members with experience of mental illness (including service users and carers) provided feedback on design and interpretation throughout. The RECORD guidelines, a checklist devised for studies using routinely collected health data, were used to guide reporting.^22^

### Mental Health Diagnoses

We selected all patients with primary care-recorded diagnoses of schizophrenia or other related psychoses (henceforth “schizophrenia”) at any point in their lifetime up until January 31, 2020. Similarly, all patients with BD were selected for the second sample. The third sample included all individuals with recurrent major depressive disorder (henceforth “MDD”), thereby ruling out patients with single depressive episodes. For comparison purposes, we obtained records for age–sex (ie matched on sex at birth and year of birth) matched people with no prior evidence of mental illness up until January 31, 2020, sampled at a 4:1 ratio against cases (henceforth referred to as “controls”). Hence, individuals could be included in more than one sample as a case if they had multiple relevant lifetime diagnoses, though never as controls. Individuals with no prior mental illness could also appear in more than one sample as a control. For the purposes of this study, diagnoses for mental health problems following January 31, 2020 were ignored. Clinical code sets for concepts and diagnoses were developed using OpenCodelists (https://www.opencodelists.org), a tool created by OpenSAFELY to improve transparency and reusability in coding curation, and are available in our online GitHub repository (https://github.com/rw251/gm-idcr/tree/master/shared/clinical-code-sets; supplementary material – table S1). Mental health diagnostic codes corresponded with ICD codes F20-29 (schizophrenia, schizotypal, and delusional disorders), F31 (bipolar affective disorder), and F33 (recurrent depressive disorder).

### Outcomes and Covariates

The primary outcome was COVID-19 related mortality, defined as all deaths reported via NHS Digital’s personal demographics service within 28 days of a positive, lab-confirmed COVID-19 test entering the primary care record. This measure is consistent with Public Health England’s definition of COVID-19 related mortality and has been used in the United Kingdom as a standard indicator for surveillance purposes.^23^ Vaccination status was determined for each individual on a monthly basis from their primary care record as either doubly vaccinated (ie, 2 or more doses by the preceding month) or not doubly vaccinated (ie, 0 doses or 1 dose). As the booster vaccination campaign only began in September 2021 in the United Kingdom, we did not account for this in this analysis.

We also explored how demographic and clinical factors affected risk of COVID-19 related mortality among people with and without SMI. Demographic data included age (in years), sex and ethnicity (Asian; Black; mixed; White; other). Index of multiple deprivation (IMD) deciles were provided as a measure of material deprivation, calculated based on patients’ residential postcode data, decile 1 representing the most deprived and decile 10 representing the least deprived.

Comorbidity data was based on recorded primary care diagnoses (lifetime, up until January 31, 2020) for the following conditions: alcohol misuse, atrial fibrillation, cancer, chronic kidney disease, chronic liver disease, chronic obstructive pulmonary disease (COPD), coronary heart disease (CHD), dementia, diabetes, epilepsy, heart failure, learning disability, multiple sclerosis, Parkinson’s disease, peripheral vascular disease, stroke, and substance (psychoactive drug) misuse. These conditions have previously all been used to predict COVID-19 mortality.^24^

### Statistical Analysis

We investigated associations between different mental illnesses and mortality due to COVID-19, adjusted for a range of demographic and clinical variables, described herein. In preparation for analysis, data validation checks were performed to exclude invalid codes and data ranges. Owing to low counts for people with BD in certain subgroup analyses, statistical disclosure control techniques (mainly rounding to base 5) have been applied to some table cells and figures where indicated. Missing sociodemographic data were imputed using multiple imputation with chained equations under the assumption that they were missing at random.

Monthly mortality rates from February 2020 through until September 30, 2021 were calculated and visualized as line charts with 95% confidence intervals (CIs). Sample denominators were calculated on a monthly basis, taking into account reductions in denominators due to deaths resulting from COVID-19 or other causes.

Separate Poisson regression models for each psychiatric diagnosis were used to generate crude and adjusted risk ratios (RRs) and 95% CIs for mortality related to (1) COVID-19 and (2) other causes. For these models, an individual’s follow up was divided into months. In each month, the outcome was death within that month. Data for 83 COVID-19 related deaths where the date of death was missing were excluded from regression analyses. Diagnoses were treated as binary variables in all analyses. To allow vaccinations to take effect, a 1-month lag was applied meaning that vaccinations were only counted in the calendar month following the month that the dose was administered. Vaccination status was the only time-updated variable, all other variables remained at the baseline values. In multivariable adjusted models, psychiatric diagnoses were combined with vaccination status and entered as interaction terms, to investigate potential differential effects of vaccination among people with mental illnesses versus controls. Sex and comorbid diagnoses (excluding psychiatric diagnoses) were included in adjusted models as binary variables, while age and IMD decile were entered as continuous variables, and ethnicity was entered as a categorical variable.

To further test the robustness of our findings, we performed a sensitivity analysis using hierarchically defined (rather than overlapping) diagnoses of BD and MDD to generate unadjusted and adjusted RRs and 95% CIs for COVID-19 related mortality. For this analysis, each case with SMI was assigned to one of three groups: schizophrenia; BD (excluding schizophrenia); or MDD, excluding schizophrenia or BD).

All statistical analyses were conducted using R version 4.0.0. Statistical tests were conducted with significance set at *p* < .05 (two-sided).

## Results

### Sample Characteristics

Table 1 describes the demographic and clinical characteristics of the 3 samples (total *N* = 967 169). These included 48 912 people with a diagnosis of schizophrenia, 13 932 with BD and 152 489 with MDD. There was a degree of overlap between people with different psychiatric diagnoses, particularly among people with BD, 82.2% (*n* = 11 459) of whom had more than one diagnosis (supplementary material - figure S2). Compared to people with BD and MDD, people with schizophrenia were more likely to be male (54.1% vs 38.4%; *x*^2^ = 3686.6, df = 1, *p* < .001) and less likely to be White (77.2% vs 82.0%; df = 4, *x*^2^ = 1694.7, *p* < .001).

**Table 1.** Baseline Sample Demographic Characteristics (as of January 31, 2020, Unless Otherwise Indicated)^a^

	Schizophrenia (*N* = 48 912)		Matched Control Group, SZ^b^ (*N* = 195 645)		BD^c^ (*N* = 13 932)		Matched Control Group, BD^b^ (*N* = 55 728)		MDD (*N*=152 489)		Matched Control Group, MDD^b^ (*N* = 609 953)	
	*n*	%	*n*	%	*n*	%	*n*	%	*n*	%	*n*	%
Sex												
Female	22 455	45.9	89 817	45.9	8340	59.9	33 360	59.9	93 212	61.1	372 848	61.1
Male	26 448	54.1	105 792	54.1	5 590	40.1	22 356	40.1	59 264	38.9	237 056	38.9
Age group												
Mean, SD	50.7	19.6	50.7	19.6	50.0	16.2	50.0	16.2	50.4	16.0	50.4	16.0
Ethnicity												
Asian	3663	7.5	16 103	8.2	820	5.9	4705	8.4	6929	4.5	50 855	8.3
Black	1576	3.2	5635	2.9	270	2.0	1744	3.1	2096	1.4	17 833	2.9
Mixed	861	1.8	2404	1.2	240	1.7	704	1.3	1590	1.0	7547	1.2
Other	4185	8.6	2 298 323 841	12.2	1055	7.1	6600	11.8	13 005	8.53	72 419	11.9
White	37 749	77.2	124 798	63.8	11 305	82.0	35 743	64.1	124 759	81.8	395 427	64.8
IMD decile												
Mean, SD	3.3	2.6	4.3	2.9	3.7	2.8	4.3	2.9	3.8	2.8	3.8	2.8

^a^Cell counts and percentages may not add up to 100% of totals due to missing data and rounding (to 1 dp).

^b^Matched on age (year of birth) and sex at birth.

^c^Counts, except total, are rounded to base 5 for disclosure control purposes.

Across the 3 samples, 773 734 people were included as age–sex matched controls; these had no prior evidence of schizophrenia, BD, or depressive disorders recorded in their records by January 31, 2020. For the 5 most commonly recorded physical comorbidities, physical illnesses were less common in controls in comparison with people with SMI, specifically: history of diabetes (13.3% vs 19.7; *x*^2^ = 5088.4, *p* < .001), cancer (9.2% vs 19.2%; *x*^2^ = 15514.0, *p* < .001), CHD (3.3% vs 5.6%; *x*^2^ = 2297.4, *p* < .001), COPD (2.6% vs 5.6%; *x*^2^ = 5238.8, *p* < .001), and stroke (2.8% vs 5.3%; *x*^2^ = 3005.7, *p* < .001). In addition, alcohol use (2.1% vs 9.5%; *x*^2^ = 30114.0, *p* < .001) and substance use (1.2% vs 8.6%; *x*^2^ = 39513.0, *p* < .001) were also less common in controls compared with people with SMI. Overall, missing sociodemographic data were evident for sex (*N* = 112, <0.01%), ethnicity (*N* = 90 344, 9.3%), and IMD decile (*N* = 1239, <0.1%).

### Changes in COVID-19 Mortality Over Time

**Fig. 1.**
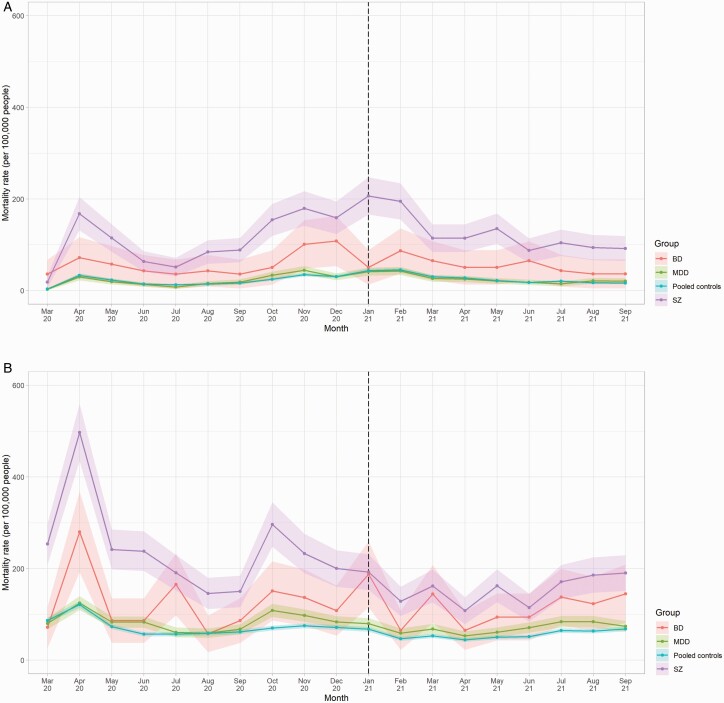
Mortality rates due to a) COVID-19 and b) other causes from March 2020 to September 2021, by diagnosis compared with pooled controls^1^. a) COVID-19 related mortality^2^. b) Mortality due to causes other than COVID-19. ^1^Dashed vertical line indicates the month following the beginning of the vaccination roll-out in the United Kingdom. February 2020 data omitted owing to low counts (disclosure control). Shaded areas represent 95% CIs. ^2I^Includes BD counts where *n* < 5 were rounded up to 5 for disclosure control purposes.

A total of 5442 people across the 3 samples died due to COVID-19 during the study observation period; 1083 had schizophrenia, 136 had BD, 926 had MDD, and 3570 had none of these. For reference, 14 423 people died of causes other than COVID-19 during the same period; 1980 had schizophrenia, 329 had BD, 2334 had MDD and 10 365 had none of these. Figure 1 shows how mortality rates due to COVID-19 and other causes varied over time. Clear peaks in COVID-19 mortality rates across all groups were observable in April 2020 (peak rates ranging from 29 to 168 deaths per 100 000 people) and November to February 2021 (34–179 deaths per 100 000). Broadly similar patterns were observable among deaths due to other causes over the same period; it is, however, notable that the first “peak” in rates in April 2020 (121–497 deaths per 100 000) occurred prior to the full roll-out of COVID-19 testing.

**Fig. 2.**
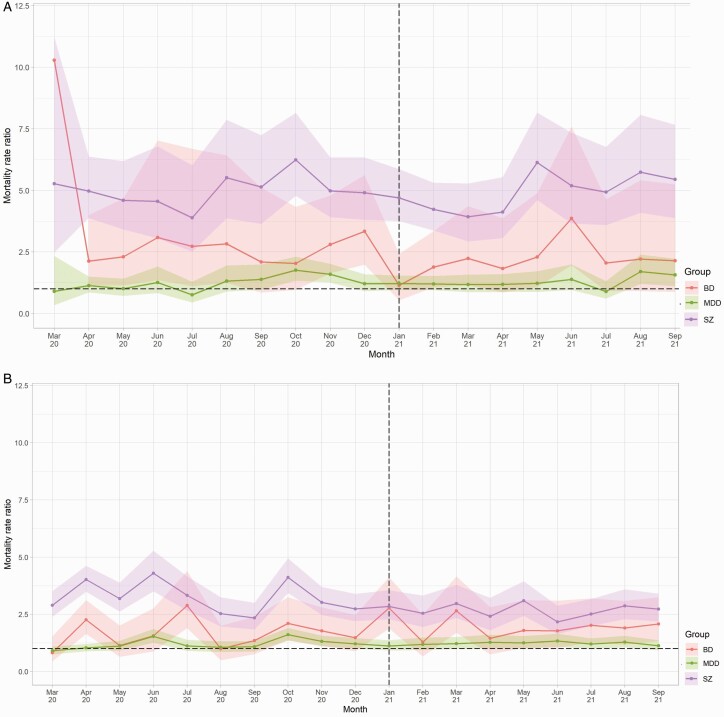
Mortality rate ratios due to a) COVID-19 and b) other causes from March 2020 to September 2021, by diagnosis compared with pooled controls^1^. a) COVID-19 related mortality. b) Mortality due to causes other than COVID-19. ^1^Dashed vertical line indicates the month following the beginning of the vaccination roll-out in the United Kingdom. Dashed horizontal line indicates RR 1.0. Shaded areas represent 95% CIs.

Compared to matched controls, vaccination rates as of September 31, 2021 were highest among people with MDD (74.0% vs 67.7%; RR 1.09, CI 1.09–1.10), followed by people with BD (72.2% vs 67.0%; RR 1.08, CI 1.05–1.10), and finally schizophrenia (65.8% vs 64.8%; RR 1.02, CI 1.00–1.03). After the vaccine roll-out began in December 2020, COVID-19 mortality rates showed significant declines across all diagnostic subgroups from January 2021 onwards, specifically people with schizophrenia (β = −13.47, *p* = .005), BD (β = −3.95, *p* = .048), and/or MDD (β = −2.97, *p* = .007). Mortality rate ratios, however, remained relatively stable (figure 2).

### COVID-19 Mortality in SMI vs General Population

Table 2 shows the RRs for the primary outcome of COVID-19 mortality associated with different SMI diagnoses. When compared against their respective matched control groups, COVID-19 mortality rates were significantly higher among people with schizophrenia (OR 3.18, CI 2.94–3.44), BD (RR 2.69, CI 2.16–3.34) and MDD (RR 1.59, CI 1.47–1.71). Relative risks of mortality due to other causes were 2.12 times higher among people with schizophrenia (CI 2.01–2.24), 2.12 times higher among people with BD (CI 1.86–2.42), and 1.32 times higher among people with MDD (CI 1.26–1.38).

**Table 2.** Relative Risk (RR) of Mortality Due to COVID-19, by Diagnosis

Diagnosis	Deaths^a^	Unadjusted^b^	Adjusted^c^
	*n* (%)	RR (95% CI)	aRR (95% CI)
Schizophrenia	1083 (2.2)	3.18 (2.94–3.44)*	1.61 (1.45–1.79)*
Matched control group for SZ	1402 (0.7)	–	–
BD	136 (1.0)	2.69 (2.16–3.34)*	1.92 (1.47–2.50)*
Matched control group for BD	207 (0.4)	–	–
MDD	926 (0.6)	1.59 (1.47–1.71)*	1.08 (0.99–1.17)
Matched control group	2352 (0.4)	–	–

^a^Includes all deaths.

^b^Includes deaths with month and year data.

^c^Includes deaths with month and year data. Adjusted for age, sex, ethnicity, deprivation (IMD decile), and preexisting comorbidities and vaccination status.

*Indicates *p* < .05.

After adjustment for vaccination, demographic and clinical variables, RRs for COVID-19 related mortality were reduced but remained significantly associated with schizophrenia (aRR 1.61, 95% CI 1.45–1.79) and BD (aRR 1.92, 95% CI 1.47–2.50), but not recurrent MDD (aRR 1.08, 95% CI 0.99–1.17). In a sensitivity analysis performed using hierarchically defined diagnoses (supplementary table S2), these results were found to be robust in the case of unadjusted associations with COVID-19 related mortality for both BD (RR 1.93, CI 1.20–3.12) and MDD (RR 1.45, CI 1.34–1.58). In the case of adjusted mortality RRs, however, neither MDD (aRR 1.05 CI 0.95–1.15) nor BD (aRR 1.54, CI 0.87–2.73) reached the threshold for statistical significance.

In multivariable adjusted regression models, COVID-19 mortality was consistently and positively associated with male gender and older age (table 3). Double vaccination was significantly associated with lower risk for COVID-19 mortality among schizophrenia and MDD, but not BD (table 3). The negative interaction effect between vaccination status and mental illness status was significant only for BD (table 3). In terms of comorbidities, history of dementia, COPD, and substance misuse all consistently showed significant associations of RR 1.5 or greater with COVID-19 related mortality regardless of diagnosis.

**Table 3.** Adjusted^a^ Multivariable Model for Relative Risk (RR) of Mortality Due to COVID-19, by Diagnosis

Variable	Schizophrenia aRR (95% CI)	BD aRR (95% CI)	MDD aRR (95% CI)
Mental illness status	1.61 (1.45–1.79)*	1.92 (1.47–2.50)*	1.08 (0.99–1.17)
Doubly vaccinated vs not vaccinated	0.76 (0.67–0.87)*	0.82 (0.59–1.16)	0.73 (0.66–0.81)*
Mental illness status* doubly vaccinated (interaction)	1.19 (0.97–1.44)	0.52 (0.28–0.97)*	0.91 (0.75–1.11)
Age (years)	1.09 (1.09–1.10)*	1.09 (1.08–1.10)*	1.10 (1.09–1.10)*
Female (ref) vs Male	1.22 (1.11–1.33)*	1.34 (1.07–1.68)*	1.32 (1.22–1.42)*
Ethnicity			
White (ref) vs Asian	0.86 (0.70–1.07)	0.96 (0.57–1.62)	1.6 (0.99–1.36)
White (ref) vs Black	1.29 (0.99–1.68)	1.78 (0.96–3.31)	1.28 (0.99–1.64)
White (ref) vs mixed	1.25 (0.78–1.99)	1.55 (0.57–4.18)	1.17 (0.75–1.82)
White (ref) vs other	1.05 (0.92–1.19)	0.93 (0.64–1.36)	1.05 (0.94–1.18)
Deprivation^b^	0.93 (0.92–0.95)*	0.92 (0.88–0.96)*	0.92 (0.91–0.93)*
Comorbidity			
Alcohol misuse	1.41 (1.18–1.70)*	1.81 (1.24–2.64)*	1.70 (1.47–1.97)*
Atrial fibrillation	1.15 (1.03–1.29)*	1.25 (0.88–1.77)	1.24 (1.11–1.38)*
Cancer	1.57 (1.44–1.70)*	1.40 (1.11–1.77)*	1.75 (1.62–1.89)*
CKD	1.19 (1.08–1.30)*	1.09 (0.82–1.44)	1.23 (1.12–1.34)*
Chronic liver disease	1.33 (0.99–1.78)	1.76 (0.94–3.29)	1.80 (1.46–2.21)*
COPD	1.56 (1.39–1.74)*	1.50 (1.11–2.02)*	1.57 (1.43–1.73)*
CHD	1.13 (1.02–1.25)*	0.96 (0.70–1.32)	1.01 (0.92–1.11)
Dementia	2.40 (2.16–2.67)*	2.81 (2.07–3.83)*	3.39 (3.07–3.73)*
Diabetes	0.99 (0.91–1.08)	1.25 (0.99–1.58)	1.06 (0.98–1.15)
Epilepsy	1.45 (1.18–1.78)*	0.82 (0.42–1.62)	1.29 (1.03–1.60*
Heart failure	1.42 (1.24–1.62)*	1.80 (1.22–2.69)*	1.50 (1.32–1.71)*
Learning disability	2.79 (2.01–3.87)*	1.87 (0.69–5.09)	3.53 (2.43–5.15)*
Multiple sclerosis	1.49 (0.71–3.12)	2.24 (0.56–0.90)*	1.73 (1.07–2.79)*
Parkinson’s disease	1.66 (1.31–2.11)*	1.75 (0.89–3.45)	1.46 (1.09–1.97)*
Peripheral vascular disease	1.26 (1.06–1.50)*	1.80 (1.17–2.78)*	1.65 (1.43–1.89)*
Stroke	1.17 (1.06–1.29)*	1.33 (0.99–1.79)	1.34 (1.22–1.47)*
Substance misuse	1.55 (1.29–1.87)*	1.52 (1.01–2.29)*	1.56 (1.31–1.87)*

^a^Adjusted for demographic variables plus atrial fibrillation, cancer, chronic kidney disease (CKD), chronic liver disease, chronic obstructive pulmonary disease (COPD), coronary heart disease (CHD), dementia, diabetes, epilepsy, heart failure, learning disability, multiple sclerosis, Parkinson’s disease, peripheral vascular disease, and stroke.

^b^Lower deciles indicate higher levels of deprivation.

*Indicates *p* < .05.

## Discussion

This study has investigated COVID-19 related disparities over time for people with schizophrenia, BD, and/or MDD in GM. Compared to age–sex matched controls, unadjusted COVID-19 mortality rates were significantly higher overall among people with any of the aforementioned mental illnesses. In the case of people with schizophrenia and/or BD, these remained significantly elevated even following adjustment for demographic factors, preexisting physical illnesses, and vaccination status.

It is well-established in the wider literature that all-cause mortality rates are higher among people with mental illness, particularly those with SMI.^3,4^ In line with several previous studies, COVID-19 mortality rates were particularly high among people with schizophrenia and/or BD.^5–7^ Moreover, the relative risk of COVID-19 mortality among people with SMI exceeded the risk of death due to other causes; indeed their relative risk of COVID-19 mortality remained consistently higher than matched controls throughout the study period, against the backdrop of raised risk of death due to other causes. The latter findings are consistent with those of Das Munshi et al.^25^ who have reported that all-cause mortality among a sample of 167 122 Londoners with a range of mental disorders and intellectual disabilities, was already higher compared with the general population, and further increased during the COVID-19 pandemic.

The reduction in RRs seen between unadjusted and adjusted models indicate that a sizeable proportion of the elevated mortality risk among people with SMI could be due to demographic factors (particularly those not used in the matching process eg, ethnicity and deprivation), alcohol and substance misuse, and underlying physical health conditions, which are known risk factors for COVID-19 mortality in the general population.^24^ Even so, controlling for demographic factors and comorbidities did not account for all of the excess risk. While a proportion of the excess risk observed could well be due to unmeasured factors—including lifestyle factors such as smoking and obesity, and the severity of physical diseases—our findings arguably add weight to the hypothesis that SMI, or its treatment, confers additional COVID-19 mortality risk independent of comorbid conditions. Unlike a previous larger, UK study of ethnic differences on COVID-19 related mortality,^26^ we found no significant effect of ethnicity. Though we included all available SMI cases and used higher-level ethnicity categories, COVID-19 deaths are (fortunately) relatively rare events, especially when considering ethnic subgroups; thus, it is possible this finding was a type 2 error resulting from insufficient statistical power.

Double vaccination was associated with significantly lower COVID-19 mortality among people with schizophrenia and MDD, though the significance threshold was not reached among people with BD (our smallest sample). The interaction between vaccination and mental illness status on COVID-19 mortality was significant only among people with BD, indicating that the protective effect of vaccination was relatively stronger in people with BD than for their matched controls. These are interesting results, which arguably warrant further investigation. Though evidence surrounding vaccination response among people with mental illness is lacking,^17^ emerging conceptual frameworks do point towards immuno-inflammatory dysregulation as a component underpinning certain mental health conditions.^27^ Mediation analysis was outside the scope of our study, and so we did not account for psychotropic medication use. This could be important given recent research suggesting that antidepressants and antipsychotics might have varying effects on COVID-19 related outcomes.^28,29^

In summary, despite population vaccination efforts that have prioritized people with SMI—and significantly higher vaccination uptake in some SMI groups—disparities still remain in COVID-19 mortality for people with SMI compared to the general population. While vaccination uptake appeared to have attenuated absolute mortality rates across the board during 2021, people with SMI continued to show higher mortality rate ratios compared to controls during the final period of our study. The results of our study therefore arguably warrant further cause for concern in the context of wider concerns that the COVID-19 pandemic may have exacerbated health inequalities for vulnerable groups, including those with mental illnesses.^25,30^ Further efforts are still required to model the complex relationships between potentially confounding, mediating and moderating factors driving increased COVID-19 mortality among people with mental illness and to identify any modifiable factors.

A major strength of this study stems from the use of electronic health records, providing our study with a large, diverse sample allowing sufficient granularity and statistical power to look at changes in mortality risks over time, while including age–sex matched controls and accounting for a range of demographic and clinical variables. In presenting findings for 3 samples of people with different mental illnesses, we are 1 of a handful of studies to have reported COVID-19 outcomes for multiple diagnoses,^25^ including distinguishing people with BD from other affective disorders, an area where more evidence is required.^7,8^ Finally, we had access to vaccination data and were able to examine mortality outcomes during the vaccination roll-out period, permitting analyses of how vaccination status affected COVID-19 related mortality among people with mental illness.

There are several limitations to our research. Though GM represents a sizeable, urban population in Northern England, this limits broader generalisability. Limited testing capacity during the first wave of coronavirus in the United Kingdom (initially testing was focused in hospitals) mean that rates of COVID-19 related mortality have likely been underestimated during this period. To preserve patient confidentiality, COVID-19 deaths and vaccinations were rounded to the nearest month preventing more fine-grained time to event analyses. Diagnoses of mental and physical illnesses were on a lifetime basis (ever/never) and derived from coded data in electronic health records (EHRs), not created specifically for research. As such, we were unable to account for the differences in the severity and burden of health problems. Furthermore, EHR diagnoses may have been under-recorded the true prevalence of SMI, leading to the potential for reduced sample sizes and/or misclassification bias. Incident diagnoses of mental illness after January 31, 2020 were ignored. This definition may have masked differential risks between those with historical (possibly resolved) illnesses and those with more recent, ongoing diagnoses.

Some people with multiple psychiatric diagnoses, particularly those with BD, were included as cases in more than one sample. Thus, a proportion of the elevated rates in people with BD and MDD may have been attributable to other (ie, BD and/or schizophrenia) diagnoses. A sensitivity analysis using stricter, hierarchical definitions of BD and MDD that excluded co-occurring schizophrenia diagnoses showed significantly elevated mortality RRs for unadjusted, but not adjusted analyses. We note, however, that the BD sample size in the sensitivity analysis performed was much reduced (by over two-thirds) and that the direction and size of the differences between unadjusted and adjusted RRs looked proportionate with those presented in the main analyses. Grouping people with affective disorders (ie, BD and MDD) was considered to avoid small sample sizes, especially for fine-grained monthly analyses; however, after consultation with our PPI group, permitting overlapping samples was, on balance, viewed as less artificial and allowed separate examination of a sizeable group of people with BD.

To date, research consistently indicates that people with mental illness, and particularly SMI, are at increased risk of poorer outcomes related to COVID-19, prompting calls for prioritizing screening, treatment and access to vaccination for such groups. Our study confirms that people with schizophrenia and BD in particular show increased risk of COVID-19 mortality compared to matched controls, even following population vaccination efforts. These findings strengthen the case for addressing the life-shortening, physical health needs of people with mental illnesses and continued research into the source of vulnerability to COVID-19 to protect these groups from further health disparities.

## Supplementary Material

sbac118_suppl_Supplementary_Material
