# Correction to: Heightened COVID-19 Mortality in People With Severe Mental Illness Persists After Vaccination: A Cohort Study of Greater Manchester Residents

**DOI:** 10.1093/schbul/sbad036

**Published:** 2023-06-20

## Abstract

**Background and hypothesis:**

Previous studies show that people with severe mental illness (SMI) are at higher risk of COVID-19 mortality, however limited evidence exists regarding risk post-vaccination. We investigated COVID-19 mortality among people with schizophrenia and other SMIs before, during and after the UK vaccine roll-out.

**Study Design:**

Using the Greater Manchester (GM) Care Record to access routinely collected health data linked with death records, we plotted COVID-19 mortality rates over time in GM residents with schizophrenia/psychosis, bipolar disorder (BD) and/or recurrent major depressive disorder (MDD) from February 2020 to September 2021. Multivariable logistic regression was used to compare mortality risk (risk ratios; RRs) between people with SMI (**N=190,188**) and age-sex matched controls (**N=760,752**), adjusted for sociodemographic factors, pre-existing comorbidities and vaccination status.

**Study Results:**

Mortality risks were significantly higher among people with SMI compared with matched controls, particularly among people with schizophrenia/psychosis **(RR 3.14, CI 2.66-3.71)** and/or BD (**RR 3.17, CI 2.15-4.67**). In adjusted models, the relative risk of COVID-19 mortality decreased, though remained significantly higher than matched controls for people with schizophrenia (**RR 1.53, CI 1.24-1.88**) and BD (**RR 2.28, CI 1.49-3.49**), but not recurrent MDD (**RR 0.92, CI 0.78-1.09**). People with SMI continued to show higher mortality rate ratios relative to controls throughout 2021, during vaccination roll-out.

**Conclusions:**

People with SMI, notably schizophrenia and bipolar disorder, were at greater risk of COVID-19 mortality compared to matched controls. Despite population vaccination efforts that have prioritised people with SMI, disparities still remain in COVID-19 mortality for people with SMI.

Erratum to: Lamiece Hassan, Chelsea Sawyer, Niels Peek, Karina Lovell, Andre F Carvalho, Marco Solmi, George Tilston, Matthew Sperrin, Joseph Firth, Heightened COVID-19 Mortality in People With Severe Mental Illness Persists After Vaccination: A Cohort Study of Greater Manchester Residents, Schizophrenia Bulletin, 2022; sbac118, https://doi.org/10.1093/schbul/sbac118

An error in the COVID-19 mortality data obtained from the data provider via the Greater Manchester Care Record led to an overestimation of the number of COVID-19 deaths across all diagnostic groups. This error affected Table 2, Table 3 and Figure 1. This error was brought to the attention of the authors and a new, corrected data extract was subsequently supplied, leading to some minor changes (decreases) in the sample size (due to more recent participant opt-outs), thereby also affecting descriptions of the sample in Table 1. The corrected Tables and Figures in the main manuscript are shown below.

**Table 1:** Baseline sample demographic characteristics (as of 31^st^ January 2020, unless otherwise indicated)^1^

	Schizophrenia (N= 47,868)		Matched control group, SZ^2^ (N=191,472)		BD^3^ (N=13,615)		Matched control group, BD^2^ (N= 54,460)		MDD (N=149,814)		Matched control group, MDD^2^ (N=599,256)	
	*n*	*%*	*n*	*%*	*n*	*%*	*n*	*%*	*n*	*%*	*n*	*%*
Sex												
** Female**	22,138	46.2	88,552	46.2	8,145	59.8	33,360	59.9	91,464	61.1	365,856	61.1
** Male**	25,719	53.7	102,876	53.7	5,465	40.1	22,356	40.1	58,332	38.9	233,328	38.9
Age group												
** Mean, SD**	51.1	19.4	51.1	19.4	50.7	16.4	50.7	16.4	50.5	16.1	50.5	16.1
Ethnicity												
** Asian**	3,717	7.8	17,444	9.1	840	6.1	4,962	9.1	6,929	4.8	55,736	9.3
** Black**	1,558	3.3	6,004	3.1	265	2.0	1,767	3.2	2,096	1.4	20,257	3.4
** Mixed**	890	1.9	2,754	1.4	225	1.7	809	1.5	1,590	1.2	8,672	1.5
** Other**	1,520	3.2	9,881	5.2	390	2.9	2,732	5.0	13,005	2.9	30,482	5.1
** White**	38,057	79.5	125,806	65.7	11,440	84.0	36,303	66.7	124,759	84.4	398,339	66.5
IMD decile												
** Mean, SD**	3.4	2.7	4.3	3.0	3.7	2.8	4.3	3.0	3.8	2.8	4.3	3.0

^1^Cell counts and percentages may not add up to 100% of totals due to missing data and rounding (to 1dp).

^2^Matched on age (year of birth) and sex at birth.

^3^Counts, except total, are rounded to base 5 for disclosure control purposes.

**Table 2:** Relative risk (RR) of mortality due to COVID-19, by diagnosis

	Deaths^1^	Unadjusted^2^	Adjusted^3^
*Diagnosis*	*n (%)*	*RR (95% CI)*	*aRR (95% CI)*
**Schizophrenia**	248 (0.5)	3.14 (2.66-3.71)*	1.53 (1.24-1.88)*
**Matched control group for SZ**	316 (0.2)	-	-
**BD**	46 (0.3)	3.17 (2.15-4.67)*	2.28 (1.49-3.49)*
**Matched control group for BD**	59 (0.1)	-	-
**MDD**	222 (0.1)	1.42 (1.21-1.66)*	0.92 (0.78-1.09)
**Matched control group**	602 (0.1)	-	-

^1^Includes all deaths.

^2^Includes deaths with month and year data.

^3^Includes deaths with month and year data. Adjusted for age, sex, ethnicity, deprivation (IMD decile) and pre-existing comorbidities and vaccination status.

* Indicates p<.05

**Table 3:** Adjusted^1^ multivariable model for relative risk (RR) of mortality due to COVID-19, by diagnosis

Variable	Schizophrenia aRR (95% CI)	BD aRR (95% CI)	MDD aRR (95% CI)
Mental illness status	1.53 (1.24-1.88)*	2.28 (1.49-3.49)*	0.92 (0.78-1.09)
Doubly vaccinated vs not vaccinated	0.23 (0.15 -0.36)*	0.26 (0.11-0.66)*	0.20 (0.14-0.28)*
Mental illness status *doubly vaccinated (interaction)	0.92 (0.48 -1.76)	0.24 (0.03-2.14)	1.60 (0.93-2.75)
Age (years)	1.09 (1.08-1.10)*	1.10 (1.08-1.12)*	1.09 (1.08-1.10)*
Female (ref) vs Male	1.33 (1.10-1.56)*	1.40 (0.94-2.10)	1.52 (1.32-1.76)*
Ethnicity			
White (ref) vs Asian	0.96 (0.64-1.44)	Insufficient data	1.40 (1.07-1.83)*
White (ref) vs Black	0.63 (0.30-1.32)	Insufficient data	0.26 (0.10-0.69)*
White (ref) vs mixed	0.71 (0.18-2.79)	Insufficient data	0.39 (0.10-1.57)
White (ref) vs other	0.69 (0.37-1.28)	Insufficient data	0.90 (0.61-1.33)
Deprivation^2^	0.91 (0.88-0.94)*	0.87 (0.80-0.93)*	0.92 (0.90-0.95)*
Comorbidity			
Alcohol misuse	1.20 (0.81-1.77)	1.42 (0.64-3.10)	1.11 (0.79-1.55)
Atrial fibrillation	1.11 (0.87-1.41)	2.23 (1.33-3.74)*	1.17 (0.94-1.45)
Cancer	1.96 (1.65-2.34)*	1.75 (1.17-2.63)*	2.28 (1.97-2.65)*
CKD	1.40 (1.16-1.69)*	1.27 (0.81-2.00)	1.43 (1.21-1.70)*
Chronic liver disease	1.72 (1.00-2.97)*	2.79 (0.98-7.94)	1.65 (1.07-2.54)*
COPD	1.68 (1.34-2.11)*	1.56 (0.94-2.58)	1.78 (1.49-2.14)*
CHD	1.15 (0.93-1.42)	0.84 (0.49-1.43)	1.05 (0.87-1.27)
Dementia	2.51 (2.02-3.12)*	3.11 (1.90-5.11)*	3.44 (2.82-4.18)*
Diabetes	0.99 (0.83-1.19)	1.38 (0.92-2.07)	1.06 (0.91-1.23)
Epilepsy	1.17 (0.74-1.84)	0.29 (0.92-2.07)	1.28 (0.82-1.98)
Heart failure	1.38 (1.05-1.81)*	1.56 (0.83-2.93)	1.88 (1.48-2.38)*
Learning disability	3.97 (2.20-7.14)*	1.90 (0.26-13.87)	3.32 (1.57-7.03)*
Multiple sclerosis	0.95 (0.13-6.75)	Insufficient data	0.95 (0.24-3.81)
Parkinson’s disease	1.72 (1.04-2.84)*	1.58 (0.56-4.47)	1.62 (0.91-2.87)
Peripheral vascular disease	0.91 (0.61-1.36)	1.50 (0.71-3.16)	1.22 (0.90-1.66)
Stroke	1.27 (1.04-1.57)*	1.61 (1.01-2.58)*	1.21 (1.00-1.47)*
Substance misuse	0.67 (1.29-1.87)*	0.62 (0.22-1.77)	0.98 (0.63-1.52)

^1^Adjusted for demographic variables plus atrial fibrillation, cancer, chronic kidney disease (CKD), chronic liver disease, chronic obstructive pulmonary disease (COPD), coronary heart disease (CHD), dementia, diabetes, epilepsy, heart failure, learning disability, multiple sclerosis, Parkinson’s disease, peripheral vascular disease and stroke.

^2^Lower deciles indicate higher levels of deprivation.

* Indicates p<.05

**Figure 1:**
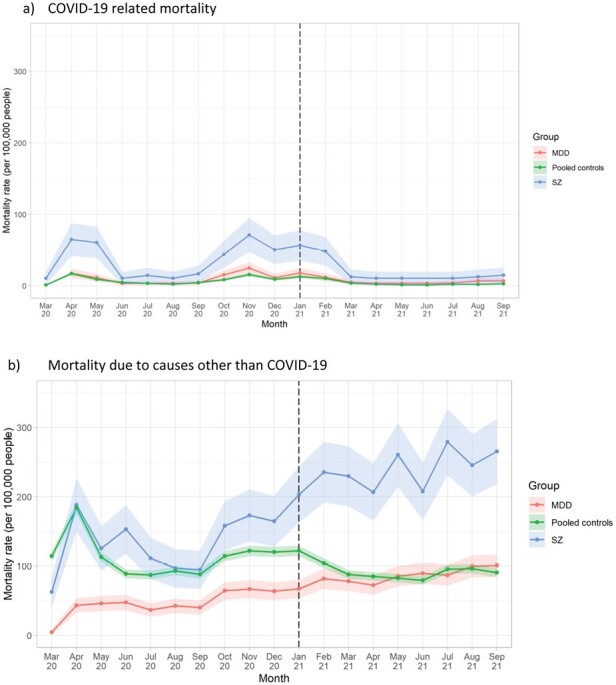
Mortality rates due to a) COVID-19 and b) other causes from March 2020 to September 2021, by diagnosis compared with pooled controls^1^. a) COVID-19 related mortality. b) Mortality due to causes other than COVID-19. ^1^Dashed vertical line indicates the month following the beginning of the vaccination roll-out in the UK. February 2020 data omitted owing to low counts (disclosure control). Shaded areas represent 95% CIs.

In the supplementary material, Table S2, Figure S1 and Figure S2 were also affected and thus a corrected version has been made available (https://doi.org/10.1093/schbul/sbac118).

**Figure 2:**
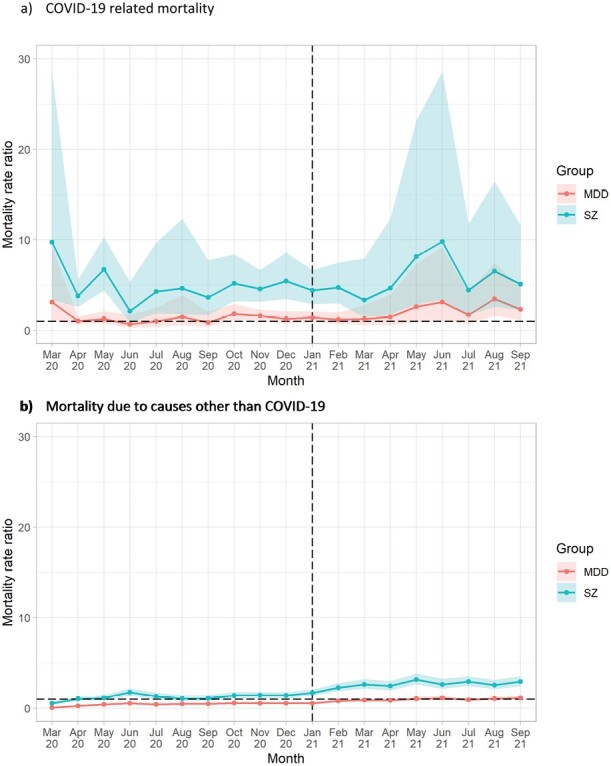
Mortality rate ratios due to a) COVID-19 and b) other causes from March 2020 to September 2021, by diagnosis compared with pooled controls^1^. a) COVID-19 related mortality. b) Mortality due to causes other than COVID-19. ^1^Dashed vertical line indicates the month following the beginning of the vaccination roll-out in the UK. Dashed horizontal line indicates RR 1.0. Shaded areas represent 95% CIs.

While the absolute number of deaths due to COVID-19 have been reduced, the differences in relative risks between groups of people with and without serious mental illness are minor. Thus, the overall findings and conclusions of the study are unchanged. The figures reported in the abstract are slightly changed as follows (see changes in bold).

As in the original version of the manuscript, when compared against their respective matched control groups, our revised analyses show that COVID-19 mortality rates were significantly higher among people with schizophrenia, bipolar disorder (BD) and major depressive disorder (MDD). Updated sensitivity analyses also show these results were robust when performed using hierarchically defined diagnoses (supplementary table S2).

In the original version of the manuscript we reported diagnosis-specific, month-by-month mortality rates (Figures 1 and 2) for schizophrenia, BD and MDD. Due to the reduced absolute numbers of deaths, we have had to omit BD-specific rates in the updated version of this manuscript. This is unfortunate, but necessary for disclosure control purposes (too many cell counts were fewer than N=5).

The originally published version of this manuscript reported that COVID-19 mortality rates showed significant linear declines after the vaccine roll-out began in December 2020. Amended analyses (see amended Figure 1) show that there were no significant linear declines in COVID-19 mortality rates.

In the revised version of the multivariate analyses examining associations between COVID-19 mortality and SMI, double vaccination was significantly associated with lower risk for COVID-19 mortality across all three diagnoses, not only schizophrenia and MDD as originally reported. There were, however, no significant interactions between vaccination status and mental illness status. In terms of comorbidities, the original version of the manuscript reported that history of dementia, COPD and substance misuse all consistently showed significant associations of RR 1.5 or greater with COVID-19 related mortality regardless of diagnosis. In the updated version, only cancer and stroke were consistently significantly associated with COVID-19 related mortality across all three diagnoses.

## Supplementary Material

sbad036_suppl_Supplementary_Material

