# Supplementary material for: Heightened COVID-19 Mortality in People With Severe Mental Illness Persists After Vaccination: A Cohort Study of Greater Manchester Residents
Source: Schizophr Bull. 2022 Aug 27;49(2):275–84. doi: 10.1093/schbul/sbac118 (PMC9452124; doi:10.1093/schbul/sbac118)
Supplement: sbac118_suppl_Supplementary_Material [file sbac118_suppl_supplementary_material.docx]

**SUPPLEMENTARY MATERIAL**

**Figure S1:** Flowchart of study sampling

**GMCR**

(N>3.2 million)

Controls sampled at 4:1

(N=54,460)

Controls sampled at 4:1

(N=191,472)

Age-sex matched controls (on year of birth) with no mental illness

Controls sampled at 4:1

(N=599,256)

All cases with MDD

(N=149,814)

All cases with BD

(N=13,615)

All cases with Schizophrenia

(N= 47,868)

**Key**

Cases

Controls sampled at 4:1

(N=200k)

**Figure S2:** Venn diagram illustrating overlap between different psychiatric diagnoses among the sample (n)

**
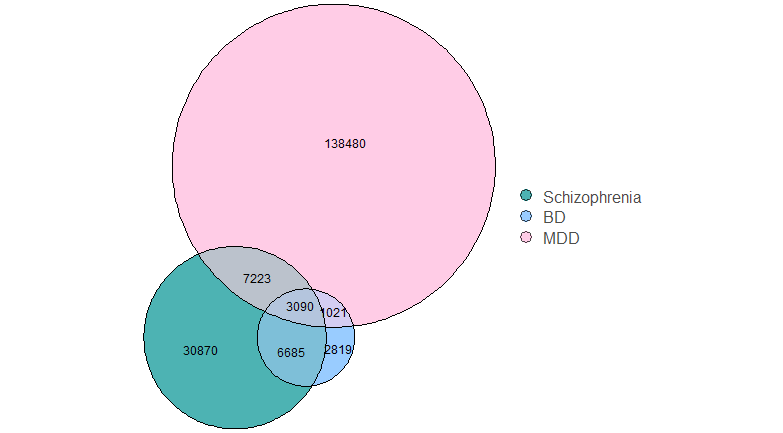
**

Table S1: URLs where Codelists may be found

| **Diagnosis** | **Code set name** | **URL^1^** |
| --- | --- | --- |
| **Mental illnesses** | | |
| Bipolar disorder | Bipolar | <https://github.com/rw251/gm-idcr/tree/master/shared/clinical-code-sets/conditions/bipolar/1> |
| Recurrent MDD | Recurrent-depressive | <https://github.com/rw251/gm-idcr/tree/master/shared/clinical-code-sets/conditions/recurrent-depressive/1> |
| Schizophrenia/ psychosis | Schizophrenia-psychosis | <https://github.com/rw251/gm-idcr/tree/master/shared/clinical-code-sets/conditions/schizophrenia-psychosis/1> |
| **Physical illnesses** | | |
| Alcohol misuse | Alcohol-problems | <https://github.com/rw251/gm-idcr/tree/master/shared/Long-term%20conditions/Substance%20Abuse> |
| Atrial fibrillation | Atrial-fibrillation | <https://github.com/rw251/gm-idcr/tree/master/shared/Long-term%20conditions/Cardiovascular> |
| Cancer | Cancer | <https://github.com/rw251/gm-idcr/tree/master/shared/Long-term%20conditions/Cancer> |
| CKD | Chronic-kidney-disease | <https://github.com/rw251/gm-idcr/tree/master/shared/Long-term%20conditions/Renal%20or%20Urological> |
| Chronic liver disease | Chronic-liver-disease | <https://github.com/rw251/gm-idcr/tree/master/shared/Long-term%20conditions/Gastrointestinal> |
| COPD | COPD | <https://github.com/rw251/gm-idcr/tree/master/shared/Long-term%20conditions/Respiratory> |
| CHD | Coronary-heart-disease | <https://github.com/rw251/gm-idcr/tree/master/shared/Long-term%20conditions/Cardiovascular> |
| Dementia | Dementia | <https://github.com/rw251/gm-idcr/tree/master/shared/Long-term%20conditions/Psychiatric> |
| Diabetes | Diabetes | <https://github.com/rw251/gm-idcr/tree/master/shared/Long-term%20conditions/Endocrine> |
| Epilepsy | Epilepsy | <https://github.com/rw251/gm-idcr/tree/master/shared/Long-term%20conditions/Neurological> |
| Heart failure | Heart-failure | <https://github.com/rw251/gm-idcr/tree/master/shared/Long-term%20conditions/Cardiovascular> |
| Learning disability | Learning-disability | <https://github.com/rw251/gm-idcr/tree/master/shared/Long-term%20conditions/Sensory%20Impairment%20or%20Learning%20Disability> |
| Multiple sclerosis | Multiple-sclerosis | <https://github.com/rw251/gm-idcr/tree/master/shared/Long-term%20conditions/Neurological> |
| Parkinson’s disease | Parkinsons-disease | <https://github.com/rw251/gm-idcr/tree/master/shared/Long-term%20conditions/Neurological> |
| Peripheral vascular disease | Peripheral-vascular-disease | <https://github.com/rw251/gm-idcr/tree/master/shared/Long-term%20conditions/Cardiovascular> |
| Stroke | Stroke-and-tia | <https://github.com/rw251/gm-idcr/tree/master/shared/Long-term%20conditions/Cardiovascular> |
| Substance Misuse | Psychoactive-substance-abuse | <https://github.com/rw251/gm-idcr/tree/master/shared/Long-term%20conditions/Substance%20Abuse> |

^1^ Page may include several files relevant to different code systems used by healthcare providers, including Read, ctv, EMIS and SNOMED codes. Note this is a live website and URLs may change slightly if/when updated.

Table S2: Relative risk (RR) of mortality due to COVID-19, by diagnosis – sensitivity analysis using hierarchically defined diagnoses

|  | Deaths^1^ | Unadjusted^2^ | Adjusted^3^ |
| --- | --- | --- | --- |
| *Diagnosis - hierarchical* | *n (%)* | *RR (95% CI)* | *aRR (95% CI)* |
| Schizophrenia (N=47,868) | 248 (0.5) | 3.14 (2.66-3.71)* | 1.53 (1.24-1.88)* |
| Matched control group for SZ (N=191,472) | 316 (0.2) | - | - |
| BD (N=3,840) | 18 (0.5) | 5.15 (2.57-10.52)* | 4.01 (1.89-8.54)* |
| Matched control group for BD (N=15,360) | 14 (0.1) | - | - |
| MDD (N=138,480) | 182 (0.1) | 1.32 (1.11-1.56)* | 0.88 (0.73-1.06) |
| Matched control group (N=553,920) | 550 (0.1) | - | - |

^1^ Includes all deaths.

^2^ Includes deaths with month and year data.

^3^ Includes deaths with month and year data. Adjusted for age, sex, ethnicity, deprivation (IMD decile) and pre-existing comorbidities and vaccination status.

* Indicates p<.05
