# Supplementary material for: Heightened COVID-19 Mortality in People With Severe Mental Illness Persists After Vaccination: A Cohort Study of Greater Manchester Residents
Source: Schizophr Bull. 2022 Aug 27:sbac118. doi: 10.1093/schbul/sbac118 (PMC9452124; doi:10.1093/schbul/sbac118)
Supplement: Supplementary file 1 [file sbac118_suppl_supplementary_material.docx]

**SUPPLEMENTARY MATERIAL**

**Figure S1:** Flowchart of study sampling

**GMCR**

(N>3.2 million)

Controls sampled at 4:1

(N=55,728)

Controls sampled at 4:1

(N=195,645)

Age-sex matched controls (on year of birth) with no mental illness

Controls sampled at 4:1

(N=609,953)

All cases with MDD

(N=152,489)

All cases with BD

(N=13,932)

All cases with Schizophrenia

(N= 48,912)

**Key**

Cases

Controls sampled at 4:1

(N=200k)

**Figure S2:** Venn diagram illustrating overlap between different psychiatric diagnoses among the sample (n)

Table S1: URLs where Codelists may be found

| **Diagnosis** | **Code set name** | **URL**^**1**^ |
| --- | --- | --- |
| **Mental illnesses** |  |  |
| Bipolar disorder | Bipolar | [https://github.com/rw251/gm-idcr/tree/master/shared/clinical-code-sets/conditions/bipolar/1](https://github.com/rw251/gm-idcr/tree/master/shared/clinical-code-sets/conditions/bipolar/1) |
| Recurrent MDD | Recurrent-depressive | [https://github.com/rw251/gm-idcr/tree/master/shared/clinical-code-sets/conditions/recurrent-depressive/1](https://github.com/rw251/gm-idcr/tree/master/shared/clinical-code-sets/conditions/recurrent-depressive/1) |
| Schizophrenia/ psychosis | Schizophrenia-psychosis | [https://github.com/rw251/gm-idcr/tree/master/shared/clinical-code-sets/conditions/schizophrenia-psychosis/1](https://github.com/rw251/gm-idcr/tree/master/shared/clinical-code-sets/conditions/schizophrenia-psychosis/1) |
| **Physical illnesses** |  |  |
| Alcohol misuse | Alcohol-problems | [https://github.com/rw251/gm-idcr/tree/master/shared/Long-term%20conditions/Substance%20Abuse](https://github.com/rw251/gm-idcr/tree/master/shared/Long-term%20conditions/Substance%20Abuse) |
| Atrial fibrillation | Atrial-fibrillation | [https://github.com/rw251/gm-idcr/tree/master/shared/Long-term%20conditions/Cardiovascular](https://github.com/rw251/gm-idcr/tree/master/shared/Long-term%20conditions/Cardiovascular) |
| Cancer | Cancer | [https://github.com/rw251/gm-idcr/tree/master/shared/Long-term%20conditions/Cancer](https://github.com/rw251/gm-idcr/tree/master/shared/Long-term%20conditions/Cancer) |
| CKD | Chronic-kidney-disease | [https://github.com/rw251/gm-idcr/tree/master/shared/Long-term%20conditions/Renal%20or%20Urological](https://github.com/rw251/gm-idcr/tree/master/shared/Long-term%20conditions/Renal%20or%20Urological) |
| Chronic liver disease | Chronic-liver-disease | [https://github.com/rw251/gm-idcr/tree/master/shared/Long-term%20conditions/Gastrointestinal](https://github.com/rw251/gm-idcr/tree/master/shared/Long-term%20conditions/Gastrointestinal) |
| COPD | COPD | [https://github.com/rw251/gm-idcr/tree/master/shared/Long-term%20conditions/Respiratory](https://github.com/rw251/gm-idcr/tree/master/shared/Long-term%20conditions/Respiratory) |
| CHD | Coronary-heart-disease | [https://github.com/rw251/gm-idcr/tree/master/shared/Long-term%20conditions/Cardiovascular](https://github.com/rw251/gm-idcr/tree/master/shared/Long-term%20conditions/Cardiovascular) |
| Dementia | Dementia | [https://github.com/rw251/gm-idcr/tree/master/shared/Long-term%20conditions/Psychiatric](https://github.com/rw251/gm-idcr/tree/master/shared/Long-term%20conditions/Psychiatric) |
| Diabetes | Diabetes | [https://github.com/rw251/gm-idcr/tree/master/shared/Long-term%20conditions/Endocrine](https://github.com/rw251/gm-idcr/tree/master/shared/Long-term%20conditions/Endocrine) |
| Epilepsy | Epilepsy | [https://github.com/rw251/gm-idcr/tree/master/shared/Long-term%20conditions/Neurological](https://github.com/rw251/gm-idcr/tree/master/shared/Long-term%20conditions/Neurological) |
| Heart failure | Heart-failure | [https://github.com/rw251/gm-idcr/tree/master/shared/Long-term%20conditions/Cardiovascular](https://github.com/rw251/gm-idcr/tree/master/shared/Long-term%20conditions/Cardiovascular) |
| Learning disability | Learning-disability | [https://github.com/rw251/gm-idcr/tree/master/shared/Long-term%20conditions/Sensory%20Impairment%20or%20Learning%20Disability](https://github.com/rw251/gm-idcr/tree/master/shared/Long-term%20conditions/Sensory%20Impairment%20or%20Learning%20Disability) |
| Multiple sclerosis | Multiple-sclerosis | [https://github.com/rw251/gm-idcr/tree/master/shared/Long-term%20conditions/Neurological](https://github.com/rw251/gm-idcr/tree/master/shared/Long-term%20conditions/Neurological) |
| Parkinson’s disease | Parkinsons-disease | [https://github.com/rw251/gm-idcr/tree/master/shared/Long-term%20conditions/Neurological](https://github.com/rw251/gm-idcr/tree/master/shared/Long-term%20conditions/Neurological) |
| Peripheral vascular disease | Peripheral-vascular-disease | [https://github.com/rw251/gm-idcr/tree/master/shared/Long-term%20conditions/Cardiovascular](https://github.com/rw251/gm-idcr/tree/master/shared/Long-term%20conditions/Cardiovascular) |
| Stroke | Stroke-and-tia | [https://github.com/rw251/gm-idcr/tree/master/shared/Long-term%20conditions/Cardiovascular](https://github.com/rw251/gm-idcr/tree/master/shared/Long-term%20conditions/Cardiovascular) |
| Substance Misuse | Psychoactive-substance-abuse | [https://github.com/rw251/gm-idcr/tree/master/shared/Long-term%20conditions/Substance%20Abuse](https://github.com/rw251/gm-idcr/tree/master/shared/Long-term%20conditions/Substance%20Abuse) |

^1 ^Page may include several files relevant to different code systems used by healthcare providers, including Read, ctv, EMIS and SNOMED codes. Note this is a live website and URLs may change slightly if/when updated.

Table S2: Relative risk (RR) of mortality due to COVID-19, by diagnosis – sensitivity analysis using hierarchically defined diagnoses

|  | Deaths^1^ | Unadjusted^2^ | Adjusted^3^ |
| --- | --- | --- | --- |
| *Diagnosis - hierarchical* | *n (%)* | *RR (95% CI)* | *aRR (95% CI)* |
| Schizophrenia (N=48,912) | 1,083 (2.2) | 3.18 (3.08-3.29)* | 1.61 (1.45-1.79)* |
| Matched control group for SZ (N=195,645) | 1,402 (0.7) | - | - |
| BD (N=3,534) | 25 (0.7) | 1.93 (1.20-3.12)* | 1.54 (0.87-2.73) |
| Matched control group for BD (N=14,136) | 53 (0.4) | - | - |
| MDD (N=140,989) | 764 (0.5) | 1.45 (1.34-1.58)* | 1.05 (0.95-1.15) |
| Matched control group (N=563,953) | 2,115 (0.4) | - | - |

^1^ Includes all deaths.

^2^ Includes deaths with month and year data.

^3^ Includes deaths with month and year data. Adjusted for age, sex, ethnicity, deprivation (IMD decile) and pre-existing comorbidities and vaccination status.

* Indicates p<.05
